# Obesity, hyperglycemia and endothelial function in inner city Bronx adolescents: a cross-sectional study

**DOI:** 10.1186/1687-9856-2013-18

**Published:** 2013-10-29

**Authors:** Chhavi Agarwal, Hillel W Cohen, Radhika H Muzumdar, Rubina A Heptulla, Venkat S Renukuntla, Jill Crandall

**Affiliations:** 1 The Children’s Hospital at Montefiore, Division of Pediatric Endocrinology & Diabetes, 3415 Bainbridge Ave, 10467 Bronx, NY, USA; 2 Albert Einstein College of Medicine, Jack and Pearl Resnick Campus, 1300 Morris Park Avenue, 10461 Bronx, NY, USA

**Keywords:** Obesity, Atherosclerosis, Cardiovascular disease, Endothelial function, RH-PAT, Adipocytokines, Insulin resistance, Oral glucose tolerance test

## Abstract

**Background:**

Along with the rise in obesity, cardiovascular disease (CVD) has become the major cause of death in developed countries. Although overt coronary heart disease rarely manifests during childhood, atherosclerosis can begin by the second decade of life. Therefore, identifying reliable risk markers of early vascular disease in childhood could be important. Alteration in endothelial function (EF) is an early preclinical marker of the atherosclerotic process and can be assessed non-invasively using reactive hyperemia peripheral arterial tonometry (RH-PAT). The purpose of this study was to investigate if obesity in children is associated with lower EF as measured with RH-PAT, and if obese children with impaired glucose regulation have further impairment in RH-PAT measured EF compared to obese children with normal glucose tolerance.

**Methods:**

Cardiovascular risk factors, adipocytokines and EF using RH-PAT were evaluated in lean (n = 14) and obese (n = 37) adolescents (age 12–18 years). Based on an oral glucose tolerance test, the obese group was subdivided into: obese with normal (NGT, n = 22) and obese with impaired glucose regulation (IGR, n = 15).

**Results:**

RH-PAT score was lower in obese subjects compared to lean controls (1.70 ± 0.02 vs. 1.98 ± 0.09, P = 0.02), indicating worse EF. This difference remained significant when adjusted for age, sex and ethnicity (P = 0.02). We observed a pattern of worsening EF with increasing metabolic burden, with RH-PAT scores of 1.98 ± 0.09,1.73 ± 0.08 and 1.65 ± 0.12 in the lean, obese-NGT and obese-IGR groups, respectively, p_trend_ = 0.03. Obese subjects were more insulin resistant [higher HOMA] (p = 0.03), and had higher levels of leptin (p = 0.004), hsCRP (p = 0.0004), and TNF-α (p = 0.03) compared to lean subjects. Adjusting for insulin resistance and adipocytokines substantially attenuated the obesity association with RH-PAT, suggesting that insulin resistance and inflammation may mediate the association of EF with obesity.

**Conclusions:**

Risk factors for adult cardiovascular disease, including impaired EF, insulin resistance and inflammation, are evident in obese adolescents. Whether early detection of these cardiovascular risk factors will be useful for informing interventions to prevent disease progression needs further study.

**Trial registration:**

Clinical Trials Identifier: NCT01879033

## Background

Heart disease is the leading cause of mortality among adults in the United States. The incidence of heart disease, especially coronary artery disease, is linked to increase in obesity in adults. Of concern is that the prevalence of obesity in adolescents has almost quadrupled in the last 30 years, from 5% to 18% [1]. With the rise in childhood obesity, conditions such as metabolic syndrome, insulin resistance and type 2 diabetes [2] that were seen in adults are now increased dramatically in children. It is predicted that the current generation of children in the United States, for the first time, will have shorter life expectancies than their parents [3].

Atherosclerosis is a chronic progressive condition that may begin as early as childhood and is accelerated in the presence of defined risk factors, including hypertension, dyslipidemia and dysglycemia. In the Bogalusa Heart study, 58% of obese children, aged 5–10 years, had at least one risk factor for cardiovascular disease and 25% had two or more [4]. Large scale longitudinal studies have linked childhood low density lipoprotein (LDL) cholesterol and body mass index (BMI) to atherosclerosis in adults [5,6]. To decrease cardiovascular disease in adults it may be useful to establish risk factors and begin intervention at childhood and adolescence.

Impaired endothelial function is an early marker of coronary artery disease [7] and has been shown to correlate with traditional metabolic risk factors including obesity, diabetes and total and/high density lipoprotein (HDL) cholesterol [8]. Endothelial dysfunction in coronary or peripheral vessels constitutes an independent predictor of cardiovascular events [9,10]. Given that early endothelial function changes are reversible, measurement of endothelial function can be used to evaluate subclinical vascular changes in patients at high risk for cardiovascular disease at a time when intervention may be most effective.

Although, non-invasive vascular testing such as carotid intimal medial thickness and brachial artery ultrasound are being used in research settings, there are significant limitations with the use of these technologies in the pediatric population. Reactive hyperemia- peripheral arterial tonometry (RH-PAT) has several potential advantages over traditional reactive hyperemia measurements. Specifically, RH-PAT testing is affordable and reproducible; it is also less operator dependent than assessment of blood vessel diameter using brachial artery ultrasound. RH-PAT was recently approved by the Food and Drug Administration for clinical use in cardio vascular disease risk stratification in adults. RH-PAT testing which reflects small resistance vessel reactivity uses pneumatic fingertip probes to measure arterial pulse wave amplitude at rest and in response to increased flow, thus providing a measure of endothelial dependent vasodilation. This technique assesses endothelium-derived nitric oxide as a vasodilator, as demonstrated in disease free participants where the administration of agents that block bioavailability of nitric oxide blunted the RH-PAT response [11]. In adults, RH-PAT has been shown to correlate closely with other measures of endothelial function, such as brachial artery ultrasound, as well as direct measures of coronary arterial function [12,13]. However brachial artery ultrasound and RH-PAT are not interchangeable as they provide different information about distinct aspects of vascular biology. RH-PAT has been shown to have greater correlation with glycemic parameters. It was also shown to better reflect micro vascular impairment in the early phases of metabolic vascular disease and therefore is ideal to study young adolescent population [14]. The assessment of arterial endothelial function in obese children has been done in previous studies using brachial artery ultrasound and carotid intimal medial thickening [15-17] but there is paucity of studies using RH-PAT in pediatric obesity.

The purpose of this study was to investigate if obesity in children is associated with impaired endothelial function measured by RH-PAT, and whether such RH-PAT measured endothelial function differences by obesity may be mediated by inflammation and insulin resistance. We also explored whether obese children with hyperglycemia will have further impairment in endothelial function compared with obese children having normal glucose tolerance.

## Methods

### Study population and ethical considerations

All adolescent participants in the age range of 12–18 years were recruited from the clinics at The Children’s Hospital at Montefiore located in New York City. Lean adolescents (BMI 5th-85th percentile) were recruited from adolescent clinics and obese adolescents (BMI ≥95th percentile) were recruited from Endocrinology clinics who were referred for evaluation of obesity. The clinic population was predominantly Hispanic and African American. All participants were evaluated by the same physician. Seventy five adolescents were approached, and 61 were determined to be eligible. Participants were excluded from the study if there was presence of clinically significant renal, liver and/or cardiovascular morbidity, diagnosed inborn error of metabolism and/or endocrine disorders, including primary dyslipidemia, thyroid disorders, and genetic syndromes, active or passive smoking, use of following medications: systemic glucocorticoids (those on inhaled steroids were not excluded), anti-epileptic and psychiatric medications or use of over-the-counter nutritional supplements (except multivitamins). A total of 51 participants at puberty (Tanner stage 2 or above) completed the study and except for three participants (two Caucasians, one Asian), they were Hispanics (38) or African Americans (10).

Written informed consent was obtained from the parent or guardian of each subject and also from the participant. The protocol was approved by the Albert Einstein College of Medicine Institutional Review Board (IRB).

### Study procedures

All participants completed the same testing regimen. Participants were instructed to fast from midnight before the study and advised to avoid caffeine for the preceding 24-hr period. On the morning of testing the participants presented at the clinical research center for weight, height, waist and hip circumference measurements. Waist and hip circumference were measured as per standard protocol [18]. Both circumferences were measured in the standing position and at the end of a gentle expiration. The waist to hip ratio (WHR) was calculated to assess body fat distribution. Body mass index (BMI) was calculated using CDC 2000 growth curves [19]. Blood pressure was measured according to the American Heart Association guidelines [20], using a standard Riva-Rocci sphygmomanometer with an appropriate size cuff and a stethoscope. Participants were classified as hypertensive if their systolic or diastolic blood pressure was higher than the 95th centile for age after adjustment for height.

Reactive hyperemia- peripheral arterial tonometry testing (EndoPAT, Itamar Medical, Israel) was conducted as per standard protocol. Briefly, pulse volume was measured by a finger plethysmographic device that allows isolated detection of pulsatile arterial volume changes, which are sensed by a pressure transducer and transferred to a computer where the signal is amplified, displayed and stored. Studies were performed with the participant at rest, in a comfortable, thermo-neutral environment. Fingertip probes were placed on the index finger of both hands and 5 minutes of baseline recording were obtained. Blood flow was then occluded in one arm for 5 minutes, using a standard blood pressure cuff. Recording continues in both fingers during occlusion and for 5 minutes after release of the cuff. The RH-PAT index was calculated as the ratio of the average pulse amplitude in the post-hyperemic phase divided by the average baseline amplitude, with normalization to the signal in the control arm to compensate for any systemic changes. Lower RH-PAT ratios reflect the presence of impaired endothelial function.

Immediately after RH-PAT testing, a fasting blood sample was drawn from the non-occluded arm for measurement of glucose, insulin, liver function tests, blood urea nitrogen, creatinine, lipid profile (total cholesterol, high density lipoprotein cholesterol, low density lipoprotein cholesterol, triglyceride), high sensitive C-Reactive Protein (hsCRP), leptin, adiponectin, tumor necrosis factor alpha (TNF-α) and free fatty acids (FFA). In addition, obese participants underwent an oral glucose tolerance test (OGTT) with a glucose load of 1.75 g/kg body weight. The maximum administered dose of glucose was 75 g. Categorization of glucose tolerance status was made using American Diabetes Association guidelines [21]. Homeostatic model assessment (HOMA) was calculated for all participants from glucose and insulin concentrations obtained in the fasting state [22].

### Statistical analysis

Statistical analysis was conducted using STATA version 12 software. Comparisons of cardiovascular risk factors, adipocytokines, and RH-PAT scores were made between obese adolescents and healthy lean controls. Continuous variables that met normality assumptions were expressed as mean ± SE. Differences in proportions were assessed using Chi-square test/or Fisher’s exact test as appropriate. Cardio metabolic characteristics, RH PAT ratios, insulin resistance and inflammatory markers in obese participants and controls were compared using independent samples t-tests or Mann–Whitney U tests if normality assumptions did not hold.

A three level ordered categorical variable was constructed representing lean control, obese normal glucose tolerance (NGT) and obese impaired glucose regulation (IGR) and trends among these categories were assessed for cardiovascular risk factors, adipocytokines, and RH-PAT score using Spearmen rank correlation. Additionally linear regression models were constructed with RH-PAT as outcome variable and BMI as a continuous independent variable unadjusted and adjusted for HOMA, leptin, hsCRP and TNF-α respectively in separate models. A two-tailed alpha of 0.05 was used to indicate statistical significance.

## Results

Characteristics of the study participants are shown in Table 1. There were 37 obese participants and 14 lean controls. The obese group was further sub-grouped into those with normal and those with impaired glucose regulation. ObeseNGT was defined as a fasting glucose level < 100 mg/dl and a 2 hour postprandial glucose level < 140 mg/dl. Obese IGR was defined as a fasting level ≥100 mg/dl and/or 2 hr ≥140 mg/dl using a glucose load of 1.75 g/kg body weight (max 75 g).

**Table 1:** Clinical and metabolic characteristics of lean and obese adolescents

Characteristic^*^	***Lean control (n = 14)***	***Obese***		***P for trend***
		***NGT (n = 22)***	***IGR (n = 15)***	
**AGE (years)**	14.93 ± 0.64	15.45 ± 0.0.42	15.26 ± 0.54	0.63
**Females**	64%	72%	73%	0.85
**Race/Ethnicity**				
Hispanic	71.4%	73%	80%	0.9
AA	21.4%	18%	20%	
Others	7%	9%	0%	
**Weight (Kg)**	56.34 ± 2.49	98.42 ± 5.20	101.78 ± 4.86	0.64
**WAIST (cm)**	75.98 ± 1.86	103.54 ± 3.20	110.45 ± 4.01	<0.001
**BMI (kg/m**^**2**^**)**	22.69 ± 0.77	36.37 ± 1.89	37.44 ± 1.25	<0.001
**SYSBP (mmHg)**	117.50 ± 3.31	118.14 ± 2.18	111.53 ± 2.24	0.19
**DIASBP (mmHg)**	70.71 ± 2.41	68.31 ± 3.86	71.47 ± 2.47	0.99
**Fasting Glucose (mg/dl)**	86.9 ± 4.00	82.36 ± 1.53	101.33 ± 4.51	0.001
**HbA1C (%)**	5.55 ± 0.16	5.61 ± 0.09	5.77 ± 0.13	0.39
**HOMA-IR**	1.75 ± 0.65	4.22 ± 0.56	10.42 ± 0.43	<0.001
**TG (mg/dl)**	72.50 (53–97)	81 (61–122)	97 (62–164)	0.09
**HDL (mg/dl)**	50.5 (41–58)	41.5 (37–46)	40 (36–52)	0.21

Legend –AA—Africo-American, SYSBP—systolic BP, DYSBP—diastolic BP, HDL -- high density lipoprotein, TG—triglycerides, HOMA-IR-- homeostatic model assessment for insulin resistance: {fasting glucose (mg/dl)}{fasting insulin(μU/ml)}/40, NGT- fasting glucose level < 100 mg/dl and a 2 hour postprandial glucose level < 140 mg/d, IGR- fasting level ≥100 mg/dl and/or 2 hr ≥140. *Values are mean ± SE, median (IQR) or percent.

Obese NGT, obese IGR and lean control participants were of similar age, sex and ethnic distribution. There was a significant trend for BMI and waist circumference among the three groups going from lean control to obese IGR (both p < 0.001) (Table 1). There was also a non-significant trend towards higher triglyceride (p = 0.09) and lower HDL levels (p = 0.21). There was no significant trend in systolic or diastolic blood pressure.

### Glucose homeostasis and insulin resistance

Whereas there was no significant difference in HbA1C (5.62 ± 0.09 vs. 5.77 ± 0.13, p = 0.34) and fasting plasma glucose values (90.05 ± 2 vs. 86.9 ± 4, p = 0.52) between the obese and lean control groups, obese subjects were more insulin resistant [higher HOMA] (6.88 ± 0.9 vs. 1.75 ± 0.65, p = 0.03), compared to lean subjects and with a significant trend among the three groups (p for trend < 0.01) (Table 1).

### Endothelial function

**Figure 1:**
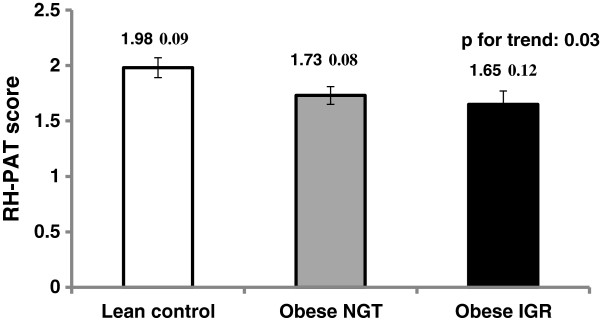
RH-PAT score (unadjusted) in lean, obese NGT & obese IGR subjects.

RH-PAT score was lower in the obese group (1.70 ± 0.02 vs. 1.98 ± 0.09, p = 0.02) compared with lean controls (p = 0.02), indicating worse endothelial function. This difference remained significant when adjusted for age, sex and ethnicity (p = 0.02). We observed a pattern of worsening endothelial function with increasing metabolic burden, with RH-PAT scores of 1.98 ± 0.09, 1.73 ± 0.08 and 1.65 ± 0.12 in the lean, obese-NGT and obese-IGR groups, respectively (p for trend =0.03) (Figure 1).

### Adipocytokines

Obese participants had higher levels of leptin (33.77 ± 2.7 vs. 18.39 ± 4.5; p = 0.004), hsCRP (4.76 ± 0.76 vs. 0.69 ± 0.24; p = 0.0004), and TNF-α (22.94 ± 4.0 vs. 13.48 ± 1.0; p = 0.03) compared with lean participants (Table 2). FFA levels (0.55 ± 0.05 vs. 0.48 ± 0.06; p = 0.38) and adiponectin levels (5.63 ± 0.42 vs 7.53 ± 1.2; p = 0.09) were similar between obese and control participants. When looked at across the three categories- lean, obese NGT and obese IGR, there were significant trends for leptin, hsCRP, TNF-alpha, and a non-significant trend for adiponectin, but no apparent trend for FFA (Table 2).

**Table 2:** Adipocytokines and RH-PAT score in lean control and obese adolescents with normal and impaired glucose tolerance test

**Adipocytokines**	***Lean control (n = 14)***	***Obese***		**Obese total (n = 37)**	P^*^	P^** ^**for trend**
		***Obese NGT (n = 22)***	***Obese IGR (n = 15)***			
**Adiponectin (μg/ml)**	7.53 ± 1.17	5.84 ± 0.51	5.17 ± 0.74	5.63 ± 0.42	0.09	0.10
**Leptin (ng/ml)**	18.39 ± 4.49	34.28 ± 3.41	32.95 ± 4.67	33.77 ± 2.71	0.004	0.02
**hsCRP (mg/l)**	0.69 ± 0.24	5.21 ± 1.11	4.16 ± 0.98	4.76 ± 0.76	0.0004	0.001
**TNF-α (pg/ml)**	13.48 ± 1.03	20.07 ± 5.05	27.12 ± 6.69	22.94 ± 4.03	0.03	0.006
**FFA (mmol)**	0.48 ± 0.06	0.60 ± 0.09	0.50 ± 0.04	0.55 ± 0.05	0.38	0.67
**RH-PAT score**	1.98 ± 0.09	1.73 ± 0.08	1.65 ± 0.12	1.70 ± 0.06	0.02	0.03

Legend -- CRP -- C-reactive protein, TNF-α—tumor necrosis factor, FFA- free fatty acids, RH-PAT -Peripheral arterial tonometry-hyperemic response. Values are mean ± SE, p*- lean vs. obese using independent t test, p**- p for trend using spearman correlation.

### Associations of BMI with RH-PAT

When adjusted for insulin resistance (HOMA-IR) or adipocytokines (leptin, hsCRP, TNF-α) the β coefficient for BMI was attenuated in each instance >50%; consistent with both insulin resistance and inflammation mediating the association of BMI with endothelial function (Table 3). Using Spearman’s correlation, RH-PAT was inversely correlated with BMI, rho = -0.32 (p = 0.02), waist circumference, rho = -0.31 (p = 0.03) and HOMA, rho = -0.32 (p = 0.04).

**Table 3:** Associations of BMI with RHPAT

	**Model 1**	**Model 2**	**Model 3**	**Model 4**	**Model 5**
**BMI**	**-0.02 p = 0.004**	**-0.009 p = 0.25**	**0.015 p = 0.20**	**-0.01 p = 0.27**	**-0.01 p =0.22**
**HOMA**		**-0.02 p = 0.20**			
**Leptin**			**-0.01 p = 0.009**		
**CRP**				**0.006 p = 0.76**	
**TNF-α**					**0.002 p = 0.59**

Values are linear regression coefficients and p values.

Model 1 unadjusted; Model 2 adjusted for HOMA; Model 3 adjusted for Leptin; Model 4 adjusted for CRP; Model 5 adjusted for TNF-α.

## Discussion

In this study, we observed worse endothelial function in adolescents with obesity compared to healthy lean controls when adjusted for age, sex and ethnicity as measured by RH-PAT. RH-PAT was inversely correlated with BMI, waist circumference, and HOMA, and showed a downward trend among lean control, obese NGT and obese IGR participants. In addition the association of BMI with RH-PAT was attenuated when adjusted for HOMA, leptin, hsCRP or TNF-alpha, consistent with mediation by these factors. These observations have important clinical relevance as alteration in endothelial function precedes anatomical changes of atherosclerosis [9].

Though other non –invasive methods to assess endothelial function using brachial artery ultrasound are well-established research tools, there are significant limitations to these techniques such as operator-dependency, technical challenges with the pediatric age group and the need for substantial training and expertise. RH-PAT offers a non-invasive method to assess endothelial function clinically in an outpatient setting, and has been shown to correlate with peripheral artery flow mediated dilatation, as well as direct measures of coronary artery endothelial function [23-25]. The findings of the current study support earlier work [26] that suggests RH-PAT could serve as a useful tool to identify children and adolescents who could develop high risk for cardio vascular disease as adults, and extends that work to a population of inner city Bronx adolescents.

Previous studies of early vascular changes in the pediatric population have reported inconsistent findings. Studies [27-29] in severely obese pre-pubertal children have failed to show correlations between carotid intimal-medial thickening with BMI or waist circumference. Other researchers [15,26] have observed significantly worse endothelial function and arterial wall stiffness in obese adolescents compared with lean controls, consistent with the current study results. One can speculate that puberty and/or time affects the progression of these changes. Longitudinal studies beginning in pre-adolescence and continuing to adulthood may be required to clarify the sequence of events that leads to atherosclerosis in children.

Obesity might promote preclinical atherosclerotic changes via a direct effect on vascular physiology. Obese children and adolescents are at an increased risk of developing cardiovascular disease in adulthood [30]. Being overweight in adolescence has been demonstrated to increase the likelihood of adult all-cause mortality, and cardiovascular disease morbidity and mortality among those overweight in adulthood [31]. Our finding of an association between leptin, hsCRP, TNF-α and HOMA with obesity supports the possibility that the increased risk other researchers have observed might be due to a prolonged exposure of arteries to the metabolic milieu [32].

Insulin has direct vasodilatory effect through stimulation of nitric oxide production in endothelial cells via PI3-K and Akt pathways [14]. In individuals with insulin resistance this action of insulin is diminished, while other effects of insulin mediated via MAP-K pathway including stimulation of migration and growth of smooth muscle cells and production of PAI-1 are intact. Therefore, individuals with insulin resistance may have abnormality in nitric oxide production by endothelial cells on the one hand and constant stimulation of pro-atherogenic changes in the vasculature in response to hyperinsulinism on the other hand, both increasing the risk of atherosclerosis [33].

Vascular inflammation is a key element in the development of cardiovascular disease [11]. In our study, obese adolescents had significantly higher acute-phase reactants and low-grade inflammation, a well-recognized component of atherosclerosis. Elevated hsCRP is consistent with a pro-inflammatory state. In adults it has also been found to predict the development of cardio vascular disease [34].

Adipose tissue secretes bioactive peptides called “adipokines” which play a major role in energy and vascular homeostasis and have been also linked to the pathogenesis of insulin resistance, metabolic syndrome, type 2 diabetes, and atherosclerosis, leading to an increased risk of cardio vascular disease [35]. Some of the adipokines analyzed in our study such as TNF-α and leptin were significantly higher in obese adolescents compared with lean controls. Elevated values in obese adolescents is consistent with an underlying low grade inflammation, as has been noted in other studies in both Hispanic and non-Hispanic obese children and adolescents [36].

Data from human studies support a direct independent pro-atherogenic role of hyperglycemia [37,38]. Our study found the lowest endothelial function in the subgroup of obese adolescents with IGR compared with obese NGT and lean controls, however the difference within the obese adolescents was less than the difference of either obese subgroup with normal controls, and we did not have sufficient statistical power with regard to a direct comparison between the obese subgroups, although we did observe statistically significant trends among the three groups. Postprandial acceleration of oxidative stress and inflammation has been observed in patients with type 2 diabetes [39] but we found no significant difference in inflammatory markers in the IGR subgroup. We cannot tell whether this reflects a true lack of difference in the population or inadequate statistical power. Larger longitudinal studies in obese adolescents with IGR beginning in preadolescent years and continuing to adulthood are needed to understand this association further.

There are some limitations to the current study. The cross-sectional design of this analysis limits our ability to infer causal relationships. Although, the RH-PAT results are not observer dependent, this method may be influenced by physiological states, such as time of the day, diet, temperature and noise [40]. However our research was performed in the early morning within a 2-h time frame, with participants in a fasted condition, in a thermo-neutral environment and at complete rest, thereby reducing the influence of these confounding factors. While the relatively small number of study participants limited our statistical power, the study group is likely representative of inner city Bronx adolescents routinely seen at our clinics.

## Conclusion

Alteration in endothelial function, insulin resistance and inflammation, all risk factors for adult cardiovascular disease are associated with obesity in adolescents. Given the current state of knowledge, the prognostic significance of the observed abnormalities identified in obese adolescents is not known, but the changes in endothelial function and the pro-inflammatory state suggest that early detrimental changes may have already begun. Further studies are warranted to investigate if interventions at these early stages informed by endothelial function measures could improve long term outcomes.

## Abbreviations

BMI: Body mass index; RH-PAT: Reactive hyperemia-peripheral artery tonometry; IGT: Impaired glucose tolerance; Hs CRP: High sensitivity C reactive protein; IL6: Interleukin 6; TNF α: Tumor necrosis factor α; FFA: Free fatty acids; HOMA: Homeostatic model assessment.

## Competing interests

The authors declare that they have no competing interests.

## Authors’ contributions

CA and JC participated in the design and conduction the study and in writing the manuscript, HWC helped with the statistical analysis and reviewed and edited the manuscript, RHM RAH VSR- helped with the results section and reviewed the manuscript. All authors read and approved the final manuscript.
